# Supplementary material for: Squamate reptiles challenge paradigms of genomic repeat element evolution set by birds and mammals
Source: Nat Commun. 2018 Jul 17;9:2774. doi: 10.1038/s41467-018-05279-1 (PMC6050309; doi:10.1038/s41467-018-05279-1)
Supplement: Supplementary file 1 — Supplementary Information [file 41467_2018_5279_MOESM1_ESM.pdf]

Supplementary Information

Squamate reptiles challenge paradigms of genomic repeat element evolution set by birds and mammals

Pasquesi *et al.*

Supplementary Note 1.

Mammal and bird genome size and transposable elements analyses. Genome size estimates based on flow cytometry analyses were retrieved for all mammal, bird and squamate reptile species available on the Genome Size database [1] (last accessed on 05 August 2017; see sheets in Supplementary Data 2) These estimates were used to calculate ranges of genome size for each lineage and for each major clade of mammals, birds and squamate reptiles (Fig. 1). For bird and mammal species, we reported estimates of the genomic TE content when *de novo* repeat annotation had previously been performed for each individual species if available. For mammal species, we report estimates available on the RepeatMasker online database[2], and we used data available in Kapusta et al. [3] for bird species (Supplementary Data 3).

Supplementary Figures

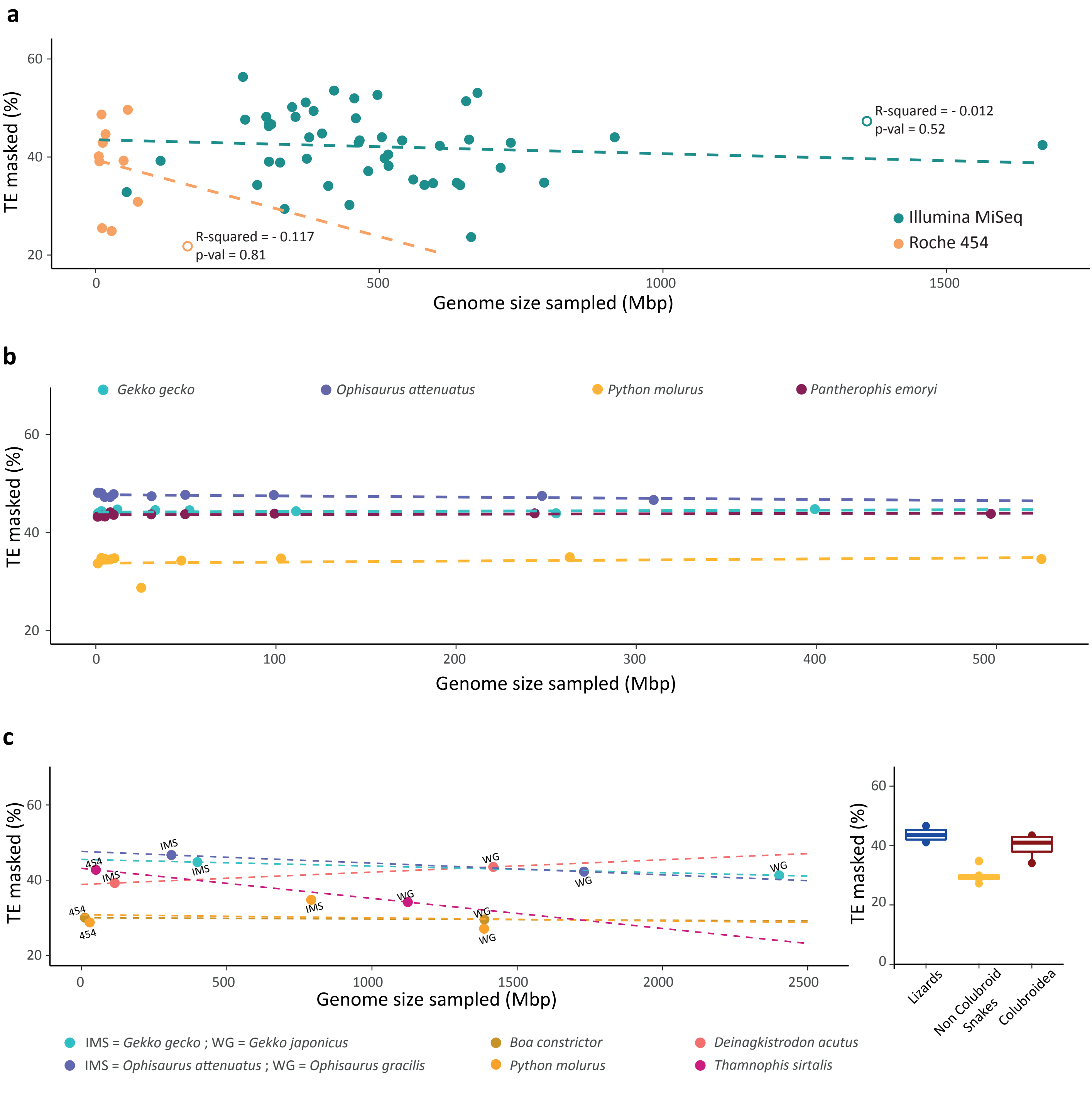

Supplementary Fig. 1. Consistency of transposable element genomic content estimates across sequencing techniques, data assembly methods and proportion of genome sampled. a) Scatterplot shows estimates of genomic TE content in relation to the amount of genome sampled from unassembled shotgun sequencing data using Roche 454 (454) and Illumina MiSeq (IMS) sequencing technologies. b) Comparison of genomic TE content estimates across subsamples of the total amount of sequence data obtained. c) Comparison of TE estimates between unassembled genomic shotgun reads and assembled whole genomes for the same species or for closely related species belonging to the same genus (left); boxplot shows the distribution of genomic TE estimates for the same species, clustered according to major squamate reptile clades (right).

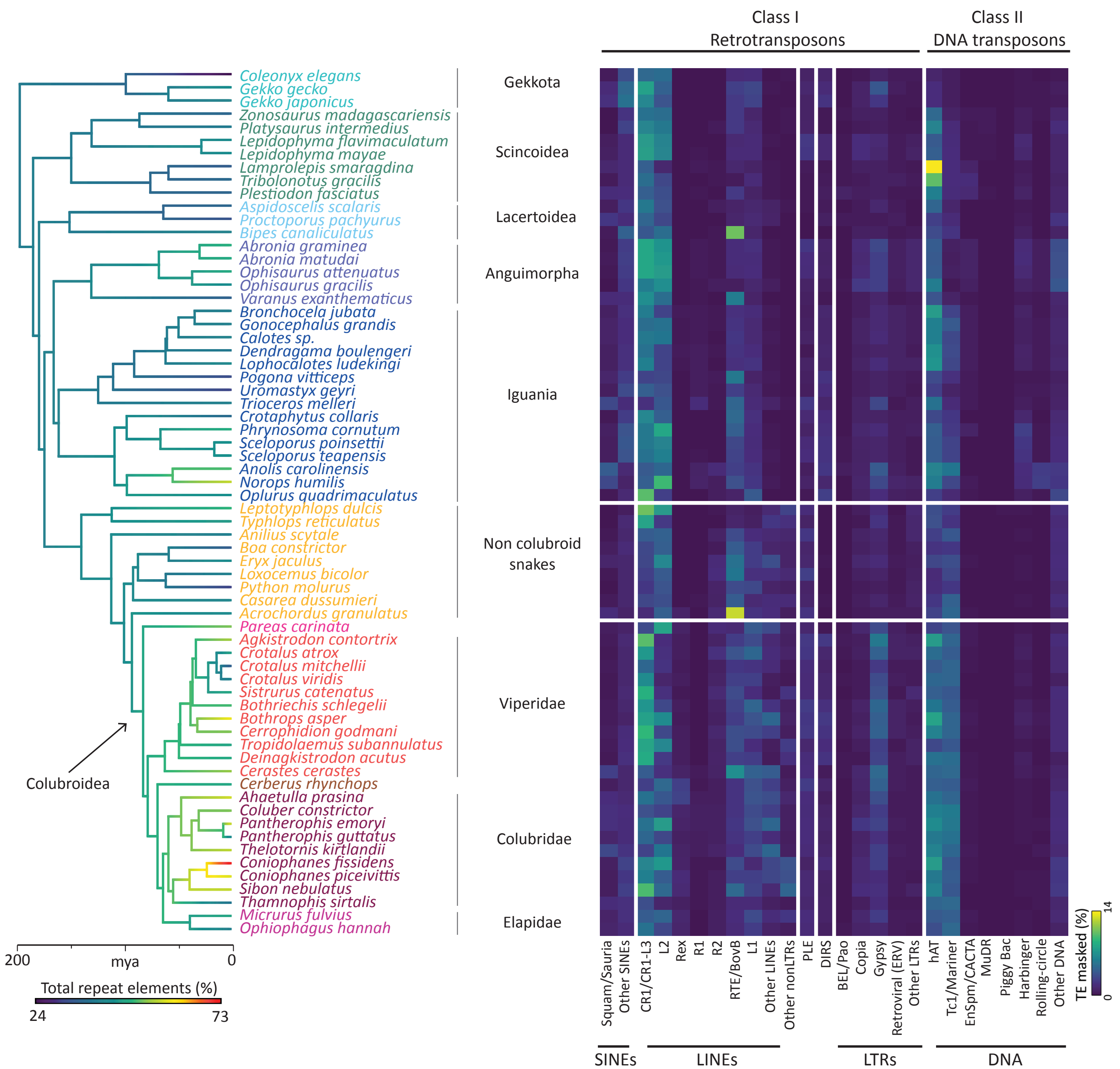

Supplementary Fig. 2. Genomic repeat element landscape for 66 squamate reptile species. Branches on the time-calibrated consensus phylogeny (left) are colored according to the estimate rates of total genomic repeat elements masked (%), allowing for an intuitive visualization of the extent of the variation in repeat element content across squamates (dark blue = lower values, red = higher values). Heat map (right) reflects variation in the relative abundance of repeat elements across 66 squamate species, and highlights both between and within clade significant differences. For example, Gypsy LTR and Tc1 DNA transposons are more abundant in the genomes of colubroid snakes than in other squamate genomes. Cells in the heat map are colored according to the color gradient: dark blue= low; yellow= high.
From left to right: Short Interspersed Nuclear Elements (SINEs); Long Interspersed Nuclear Elements (LINEs); Penelope-Like Elements (PLEs); DIRS; Long Terminal Repeat (LTR) retrotransposons, and DNA transposons.

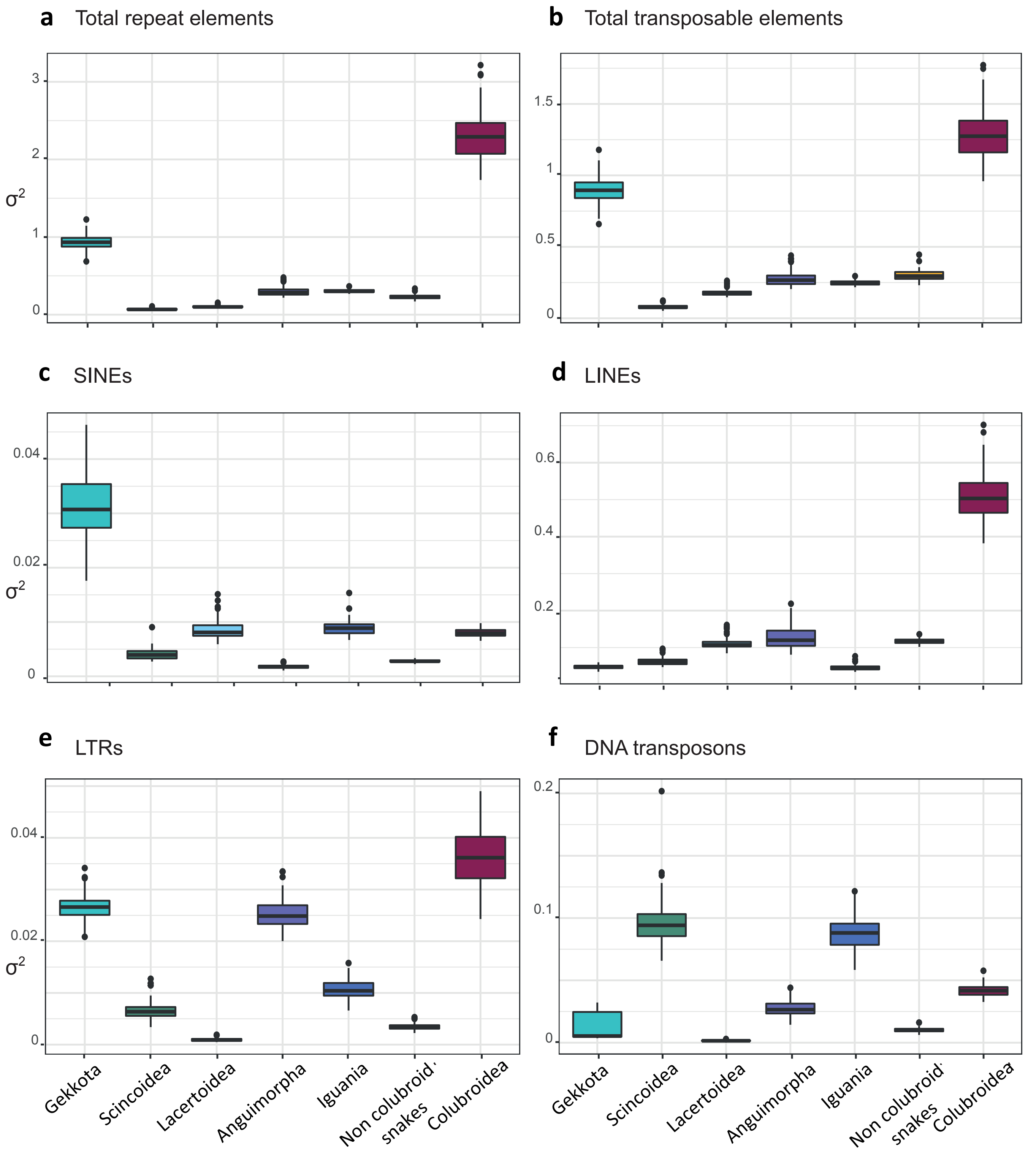

Supplementary Fig. 3. Censored rate test results for lineage-specific rates of repeat elements evolution across seven major squamate clades. Box plots represent the rate parameter (σ^2) estimates obtained across 100 trees sampled from the posterior distribution inferred from *BEAST* for the 7 major clades: gekkota(3), scincoidea(7), lacertoidea(3), anguimorpha (5), iguania (15), non colubroid snakes (9), and colubroidea (24) . The null hypothesis of a single rate of evolution for all branches was rejected for all 600 censored rate tests (100 tree for all major families of TEs and for the total repeat element and TE content; for all tests, p-values < 0.01). Results confirm that, during squamate evolution, different lineages experienced differential rates of repeat element genomic accumulation (e.g., SINEs and LTRs in colubroidea and gekkota, or LINEs in colubroidea specifically). Results are shown for (a) total repeat element content, (b) total TE content, (**c**) Short INterspersed Elements (SINEs), (d) Long INterspersed Elements (LINEs), (e) Long Terminal Repeats (LTRs), and (f) DNA transposons genomic percentages.

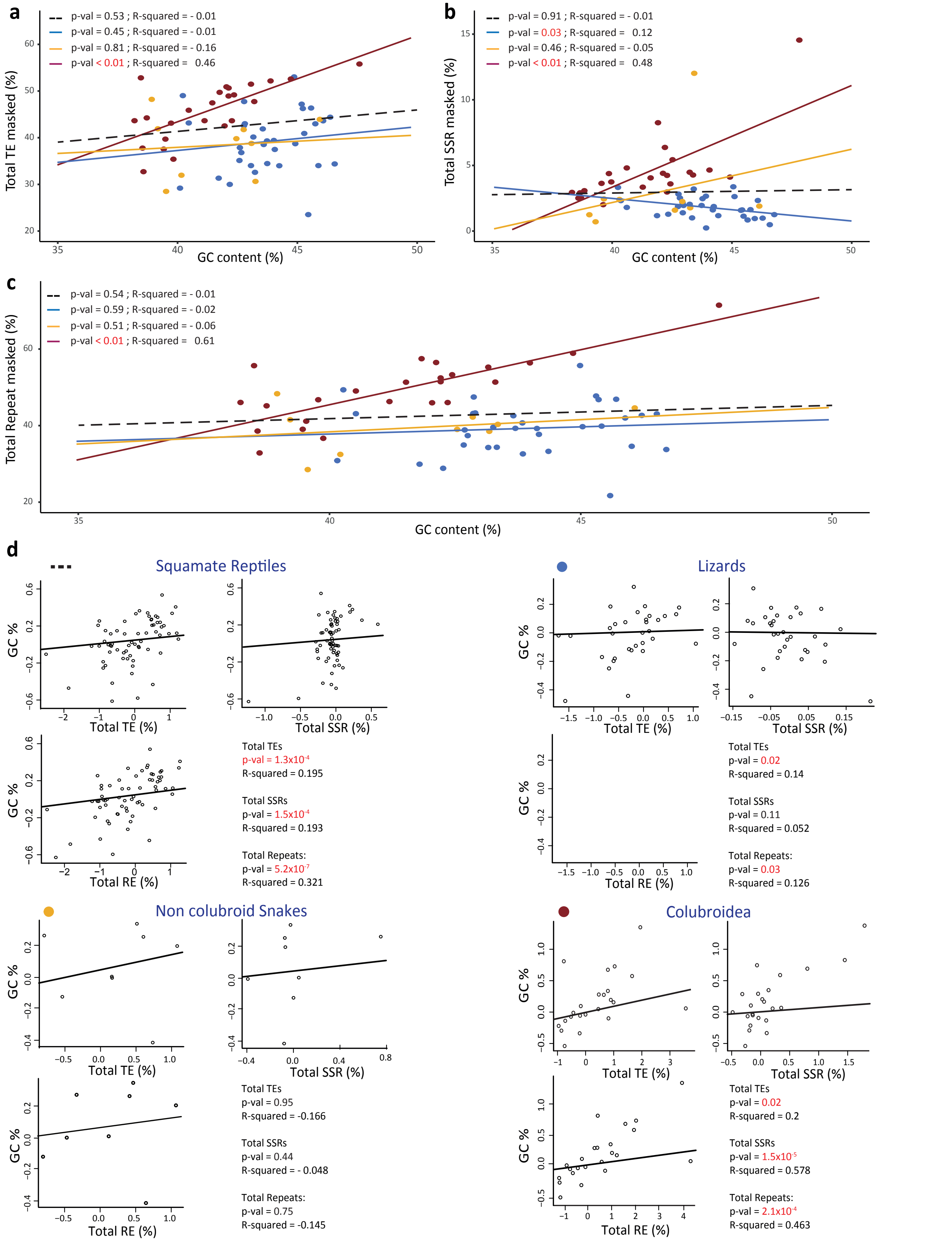

Supplementary Fig. 4. Relationship between genomic GC content (%) and genomic TE, SSR and total repeat content (%). Scatter plots reflect the relationship between average genomic GC content and genomic estimates of the major components of the repeat element landscape (%) for each clade of squamate reptiles (lizards = blue; non colubroid snakes = yellow; colubroidea = dark red). a) Analysis of the the genomic GC content and total TE content (%). b) Analysis of the the genomic GC content and microsatellite (SSR) estimates performed in RepeatMasker. c) Analysis of the the genomic GC content and total repeat element estimates. d) Phylogenetically independent contrasts (PICs) between genomic GC content, TE %, SSR % and total repeats (RE) %, for all squamates (top left) and for individual squamate lineages.

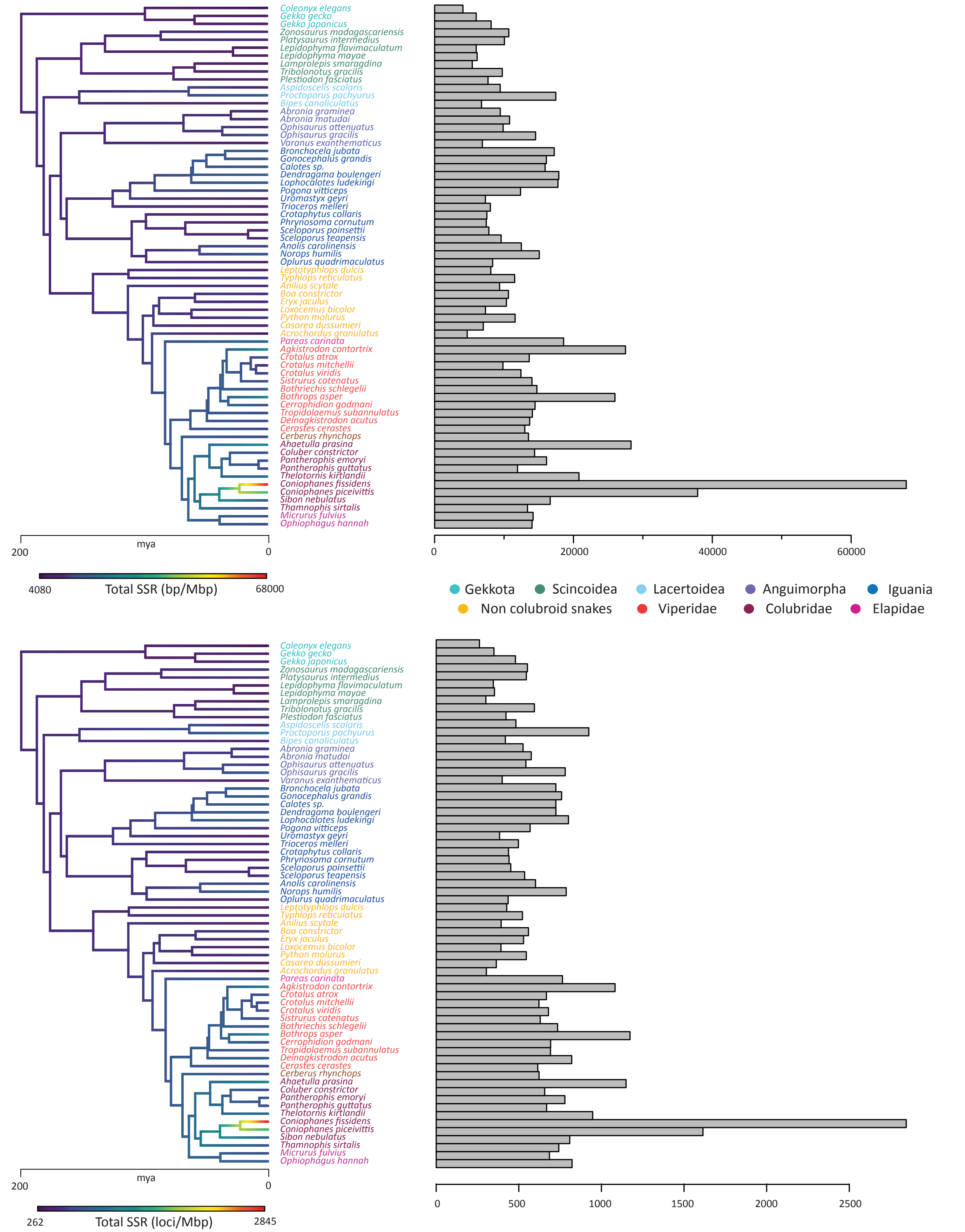

Supplementary Fig. 5. Observed total microsatellite frequencies and their lineage-specific evolutionary rates across 66 squamate species. Horizontal bar plots represent the observed total microsatellite bp/Mbp (top) and loci/Mbp (bottom) density frequencies for each squamate genome sample. Branches on the time-calibrated consensus phylogeny are colored according to the estimated rates of microsatellite evolution.

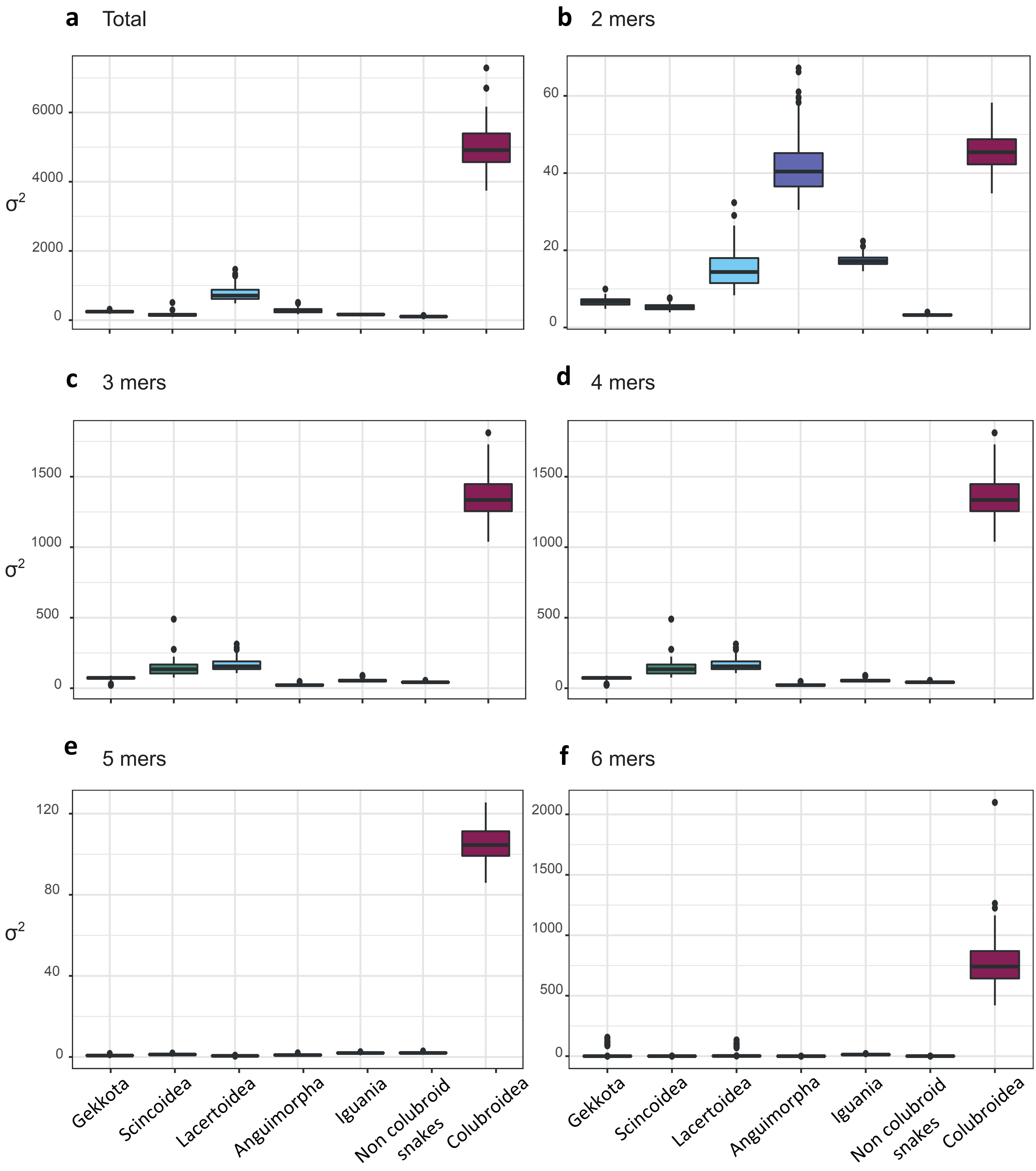

Supplementary Fig. 6. Censored rate test results for lineage-specific rates of microsatellite evolution across seven major squamate clades. Box plots represent the rate parameter (σ^2) estimates obtained across 100 trees sampled from the posterior distribution inferred from *BEAST* for the 7 major clades: gekkota(3), scincoidea(7), lacertoidea (3), anguimorpha (5), iguania (15), non colubroid snakes (9), and colubroid snakes (24). The null hypothesis of a single rate of evolution for all branches was rejected for all 600 censored rate tests (100 tree for total loci/Mbp microsatellite density estimates and for 2-6mer SSR loci/Mbp density estimates; for all tests, p-values < 0.01). Results confirm that, during squamate evolution, there has been a significant expansion of all microsatellite types among the colubroidea branch specifically compared to all other squamates. Results are shown for (a) total microsatellite content, (b) 2mer, (c) 3mer, (d) 4mer, (e) 5mer, and (f) 6mer loci/Mbp density frequencies.

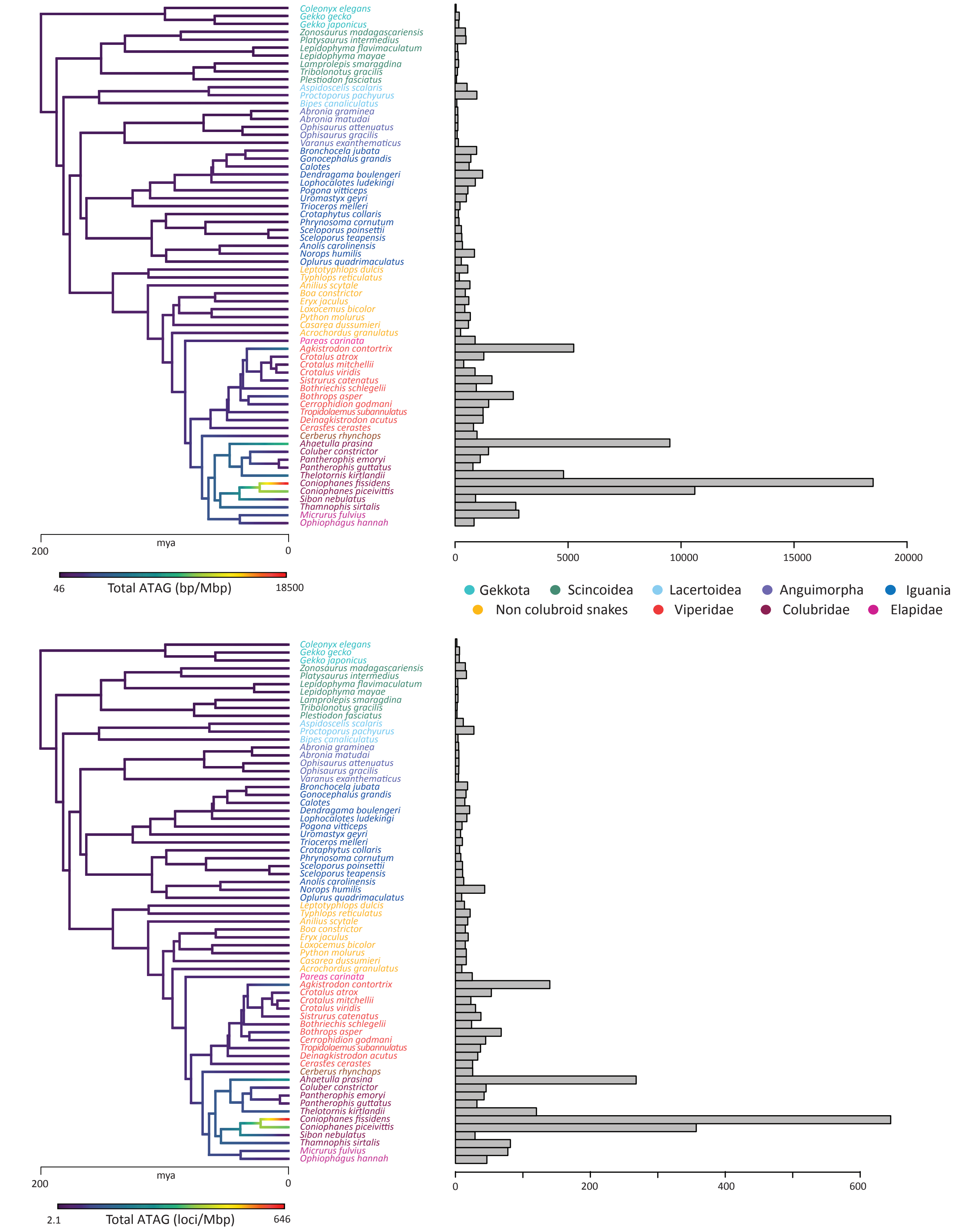

Supplementary Fig. 7. Observed ATAG microsatellite loci frequencies and their lineage-specific evolutionary rates across 66 squamate species. Horizontal bar plots represent the observed ATAG 4mer microsatellite bp/Mbp (top) and loci/Mbp (bottom) density frequencies for each squamate genome sampled. Branches on the time-calibrated consensus phylogeny are colored according to the estimated rates of microsatellite evolution.

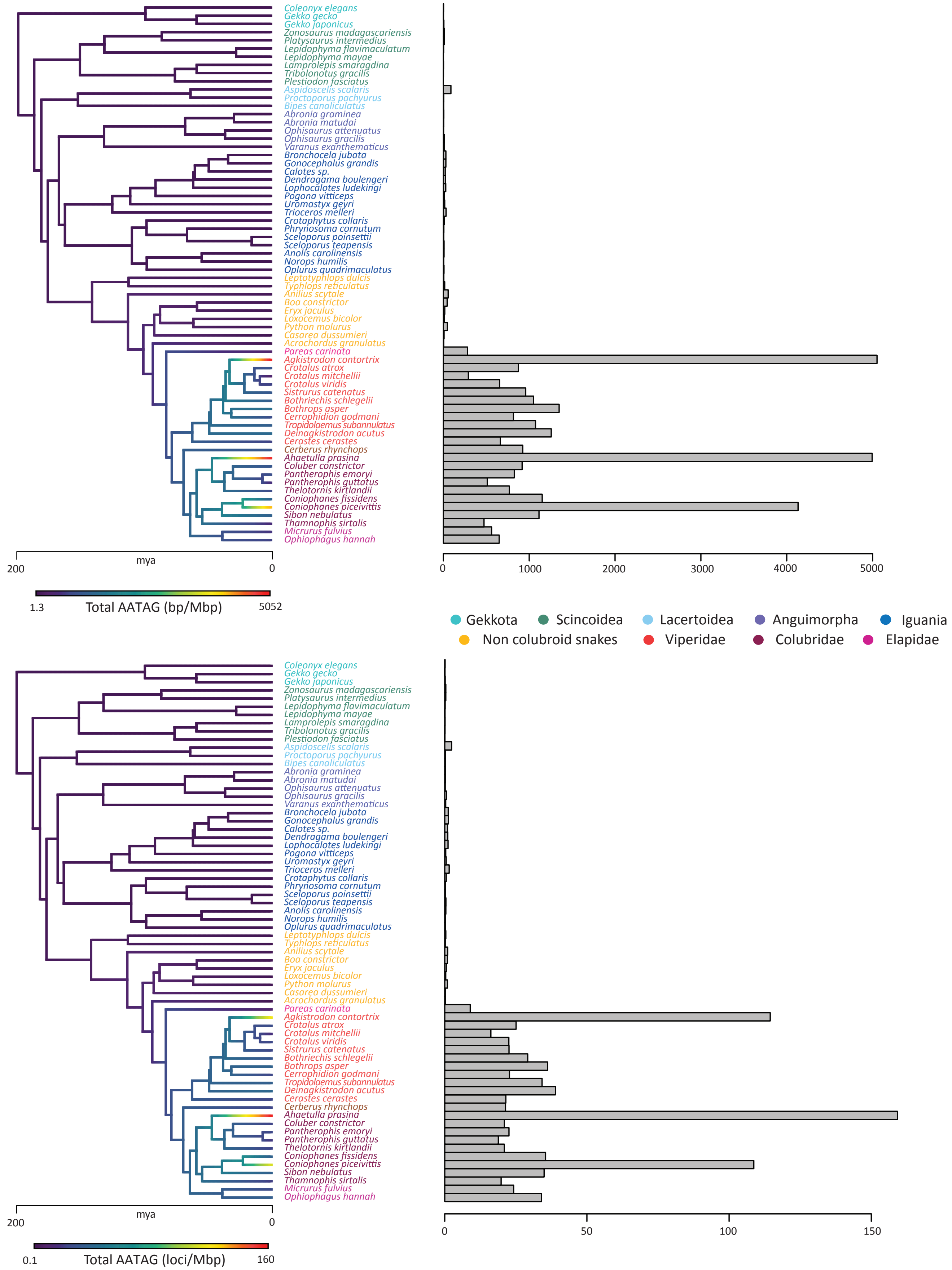

Supplementary Fig. 8. Observed AATAG microsatellite loci frequencies and their lineage-specific evolutionary rates across 66 squamate species. Horizontal bar plots represent the observed AATAG 5mer microsatellite bp/Mbp (top) and loci/Mbp (bottom) density frequencies for each squamate genome sampled. Branches on the time-calibrated consensus phylogeny are colored according to the estimated rates of microsatellite evolution.

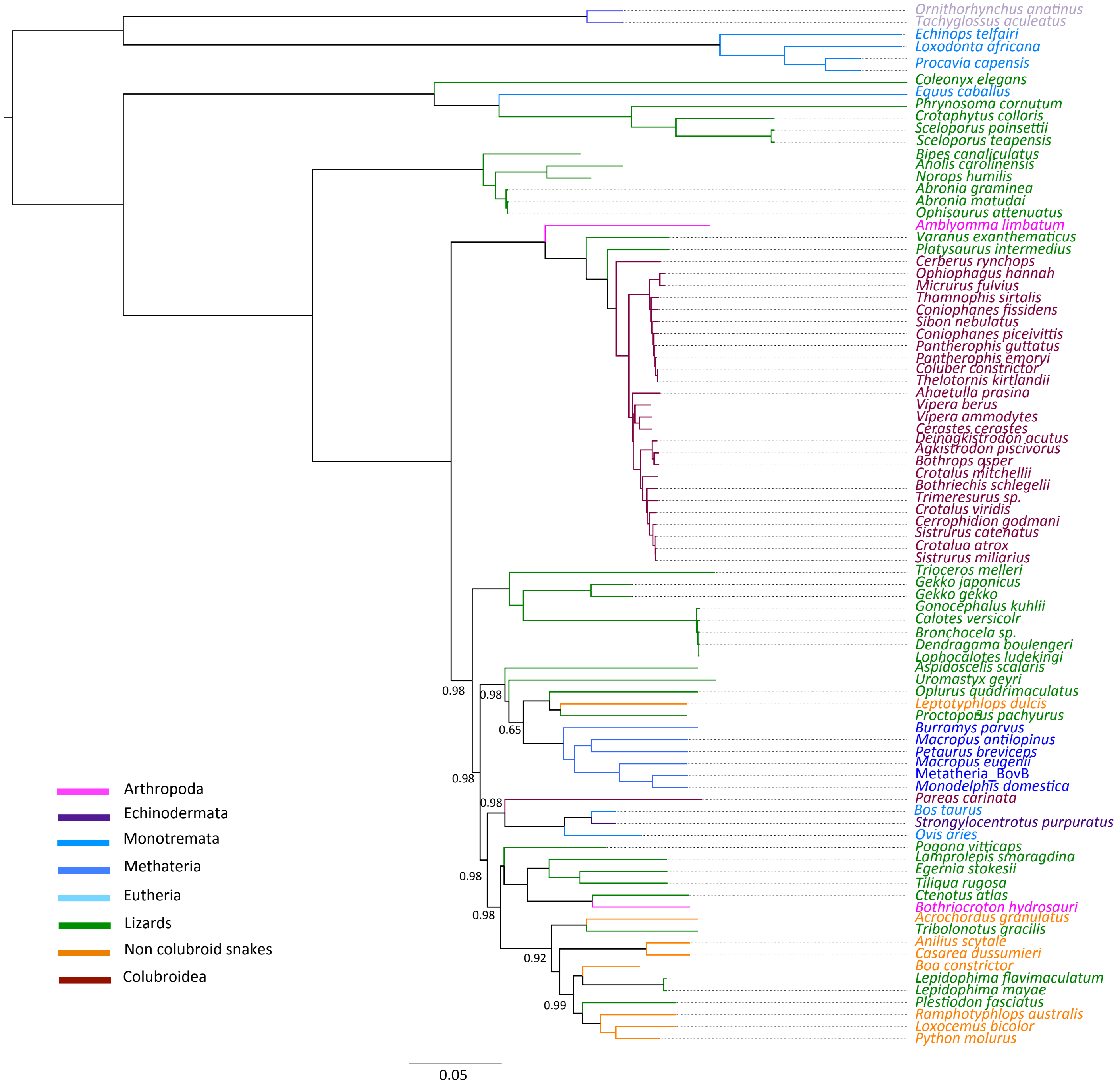

Supplementary Fig. 9a. Phylogenetic tree reconstruction of 87 metazoan BovB sequences. Bayesian phylogenetic tree was built using *BEAST2*. 141 metazoan sequences were initially aligned in Clustal W, then manually edited and curated (final alignment length of 3134bp). For displaying purposes, we pruned the RTE-2 sequences of monotremata used as outgroup to root the tree. Posterior values are reported only for nodes with posterior support < 0.99.

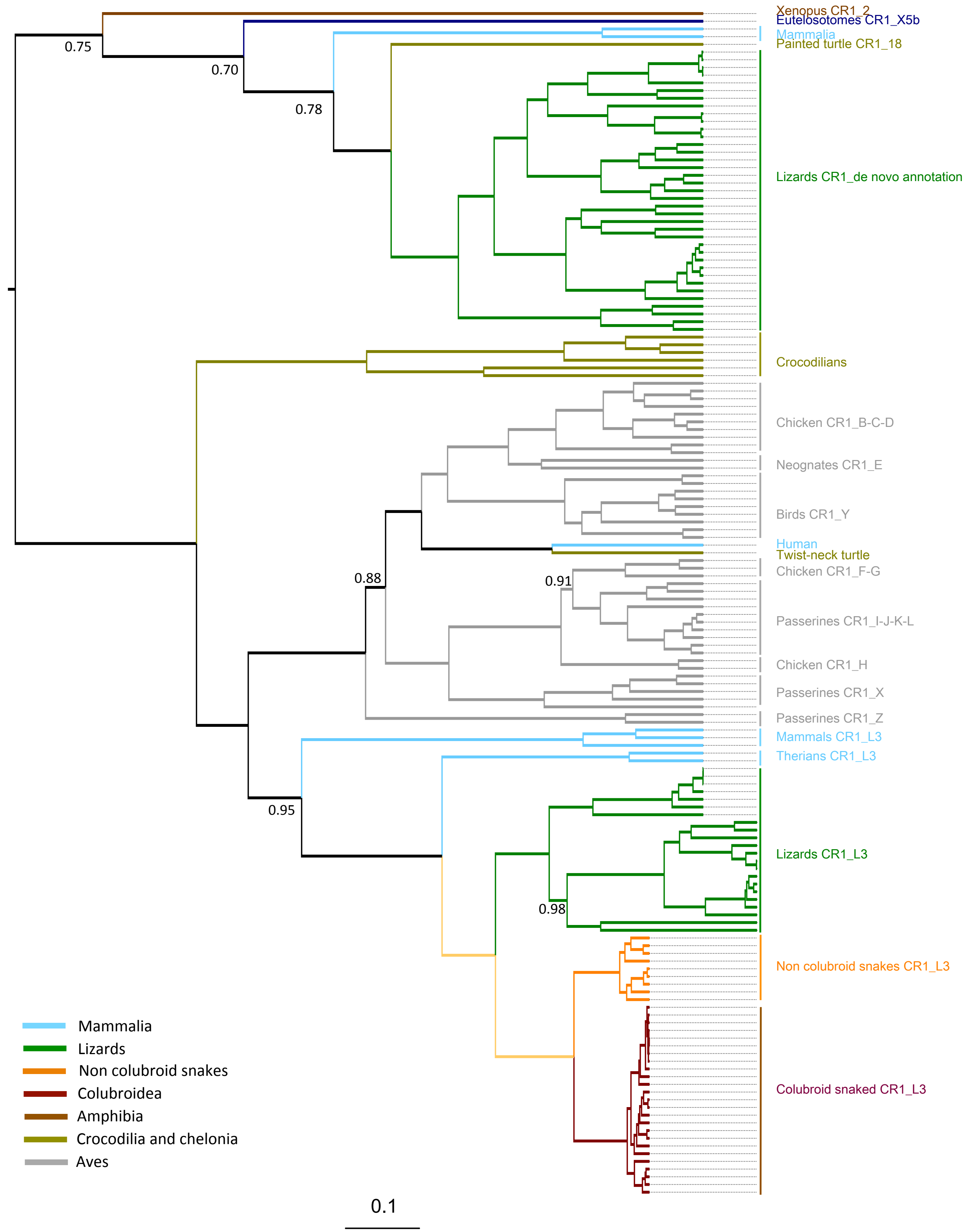

Supplementary Fig. 9b. Phylogenetic tree reconstruction of 161 metazoan CR1-L3 LINE sequences. Bayesian phylogenetic tree of the full length CR1-L3 ORF was built using BEAST2. 161 vertebrate sequences were initially aligned in ClustalW, then manually edited and curated (final alignment length of 2967bp). Posterior values are reported only for nodes with posterior support < 0.99. For displaying purposes, we pruned L2 LINEs that were used as outgroup to root the tree and extremely divergent CR1 sequences of *Xenopus tropicalis* (Xt 1a and 1b), *Danio rerio* (Dr 29) and of *Latimeria chalumnae* (Lme 1).

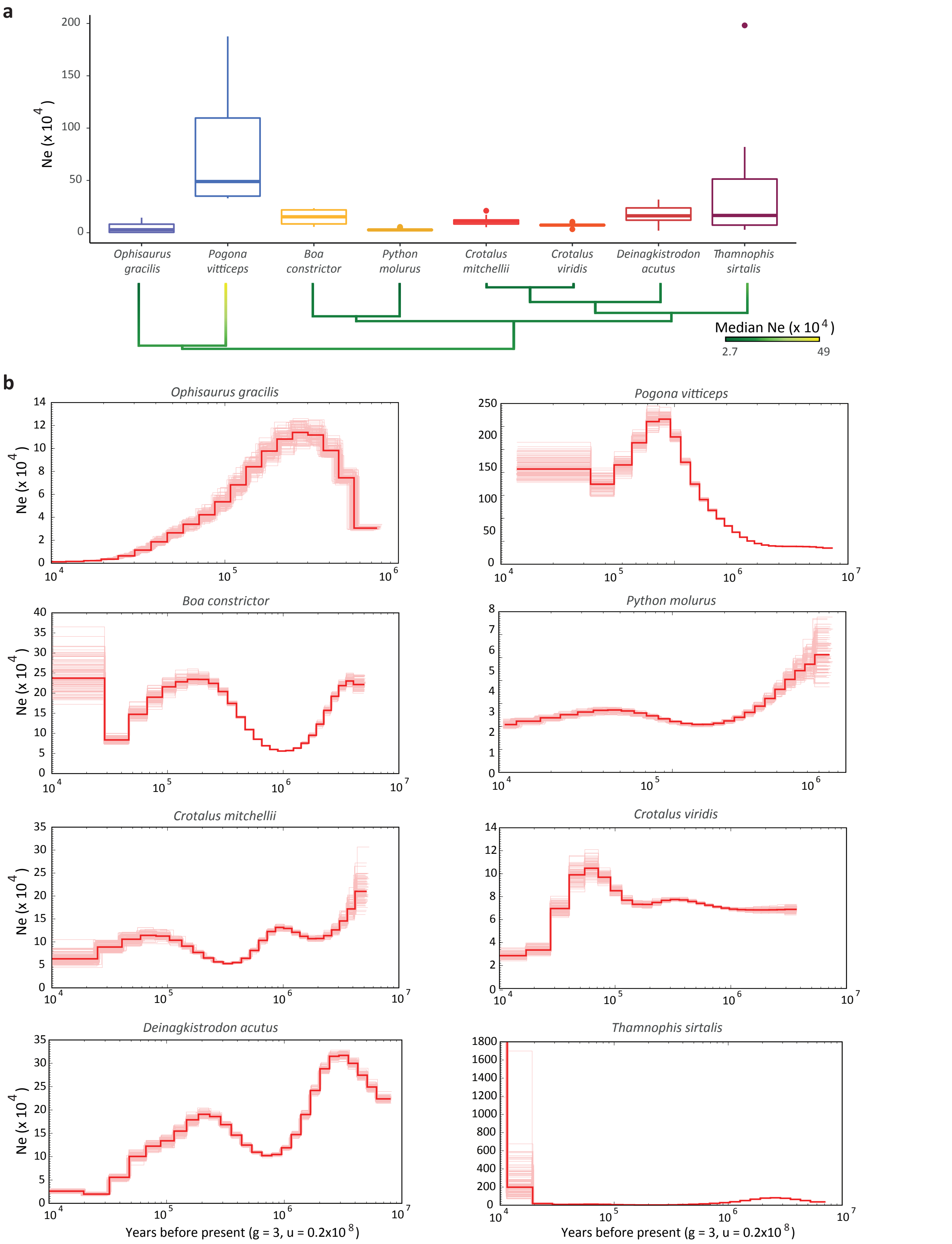

Supplementary Fig. 10 a and b. Effective population size (N_e) changes over time. a) Box plot shows the distribution of effective population size over time inferred from each genome. The first and the last time points were excluded from the input dataset. b) PSMC estimates of the changes in N_e over time inferred from each genome applying a generation time of 3 years and a mutation rate of $0.2x10^{-8}$. Plots were generated using all time points per bootstrapped sample using the *psmc_plot.pl* script.

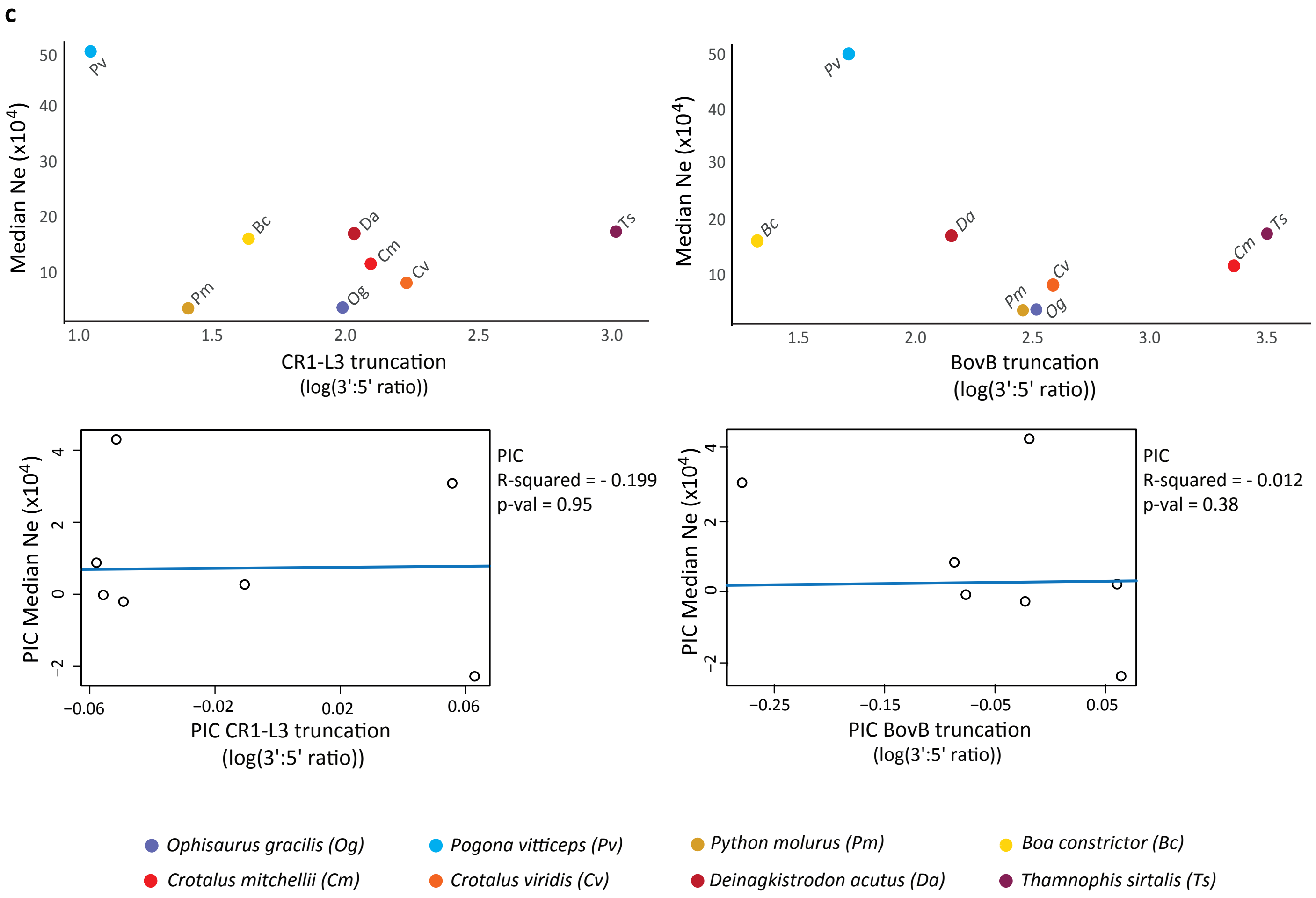

Supplementary Fig. 10c. Relationship between effective population size (N_e) and truncation. Scatter plots and associated phylogenetically independent contrasts (PICs) show a lack of a correlation between median PSMC estimates of N_e and truncation of CR1-L3 LINEs (left) and BovB LINEs (right). Adjusted R-squared values and p-values were calculated using raw, untransformed data.

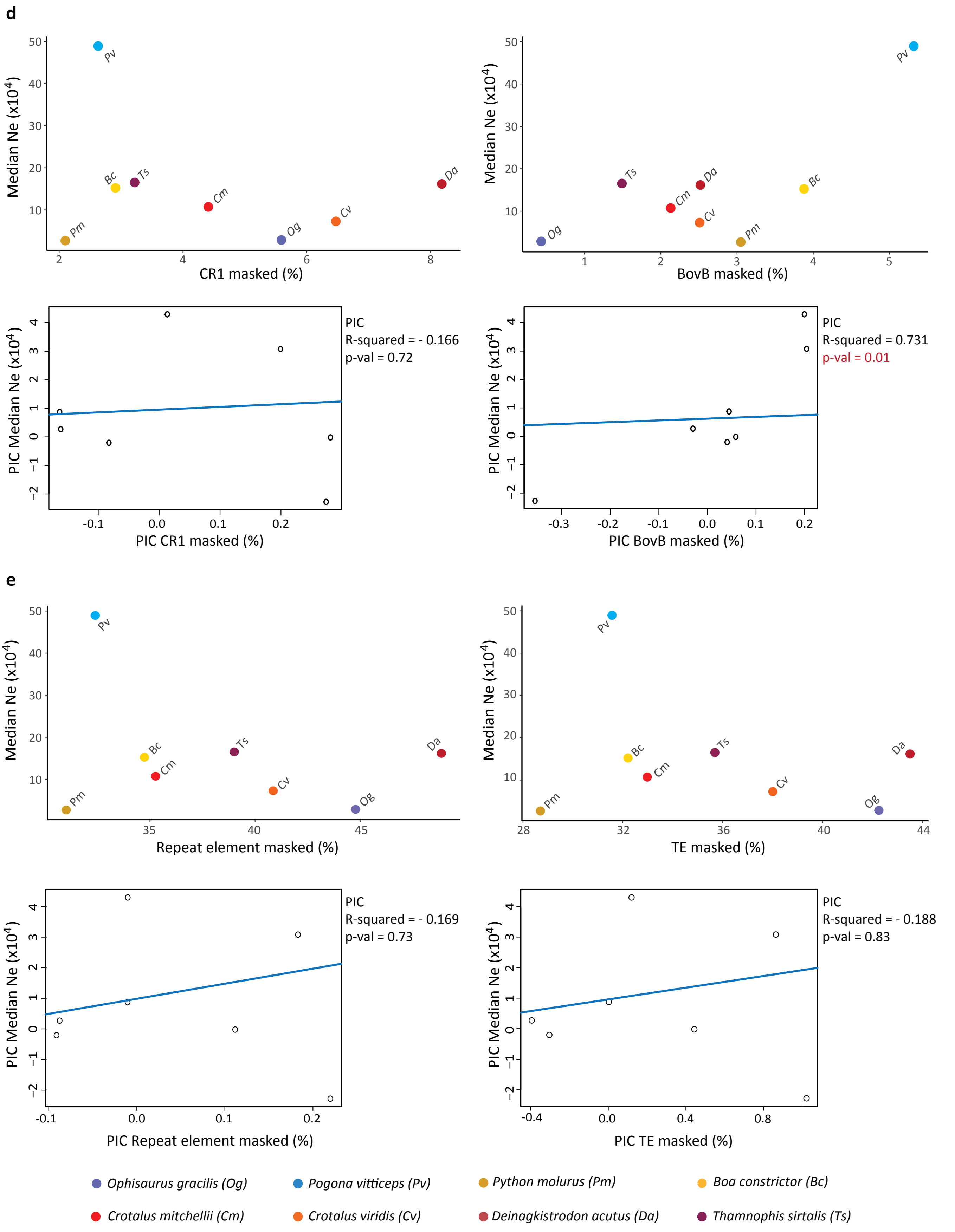

Supplementary Fig. 10 d and e. Relationship between effective population size (N_e) and genomic repeat content. d) Scatter plots and associated PICs reflect a lack of correlation between PSMC median estimates of N_e and total genomic frequency of CR1-L3 LINEs (left), and a significant positive correlation opposite to what would be expected between median N_e and BovB genomic content (right). e) Scatter plots and associated PICs reflect a lack of correlation between PSMC median estimates of N_e and total repeat element genomic content (left), and total TE content (right).

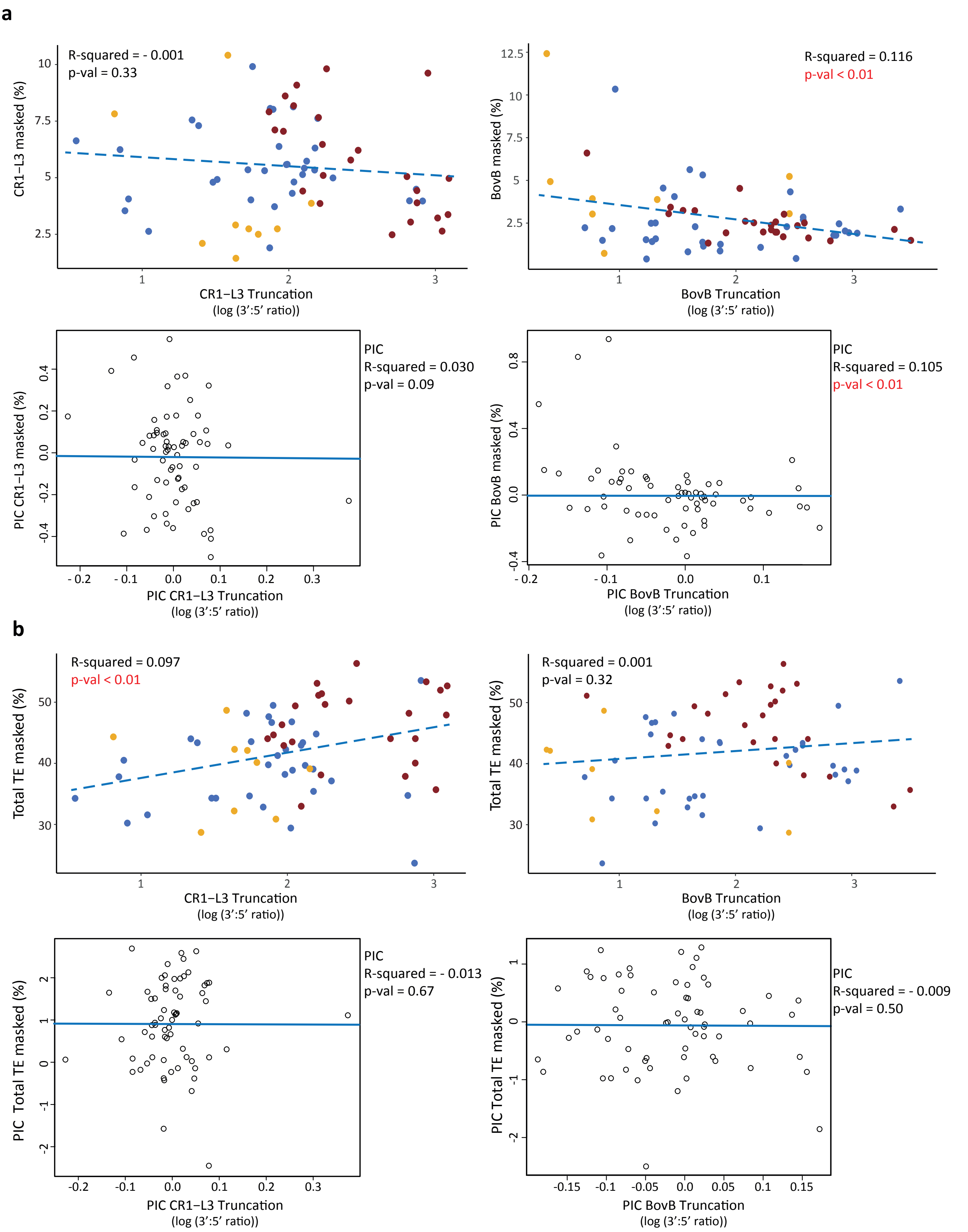

Supplementary Fig. 11. TE truncation and genomic content. Scatter plots and associated phylogenetically independent contrasts (PICs) reflect a lack of correlation between degree of truncation and total genomic content of CR1-L3 LINEs (a, left), and a weak, although significant, negative correlation between degree of truncation and total genomic content of BovB LINEs (a, right). Additionally, we report a lack of correlation between total genomic TE content and the truncation level of BovB (b, right) across squamate reptile species. For CR1-L3, we report a positive correlation that contrast model expectations. Truncation values were log-transformed prior to perform statistical tests to meet the assumption of normality.

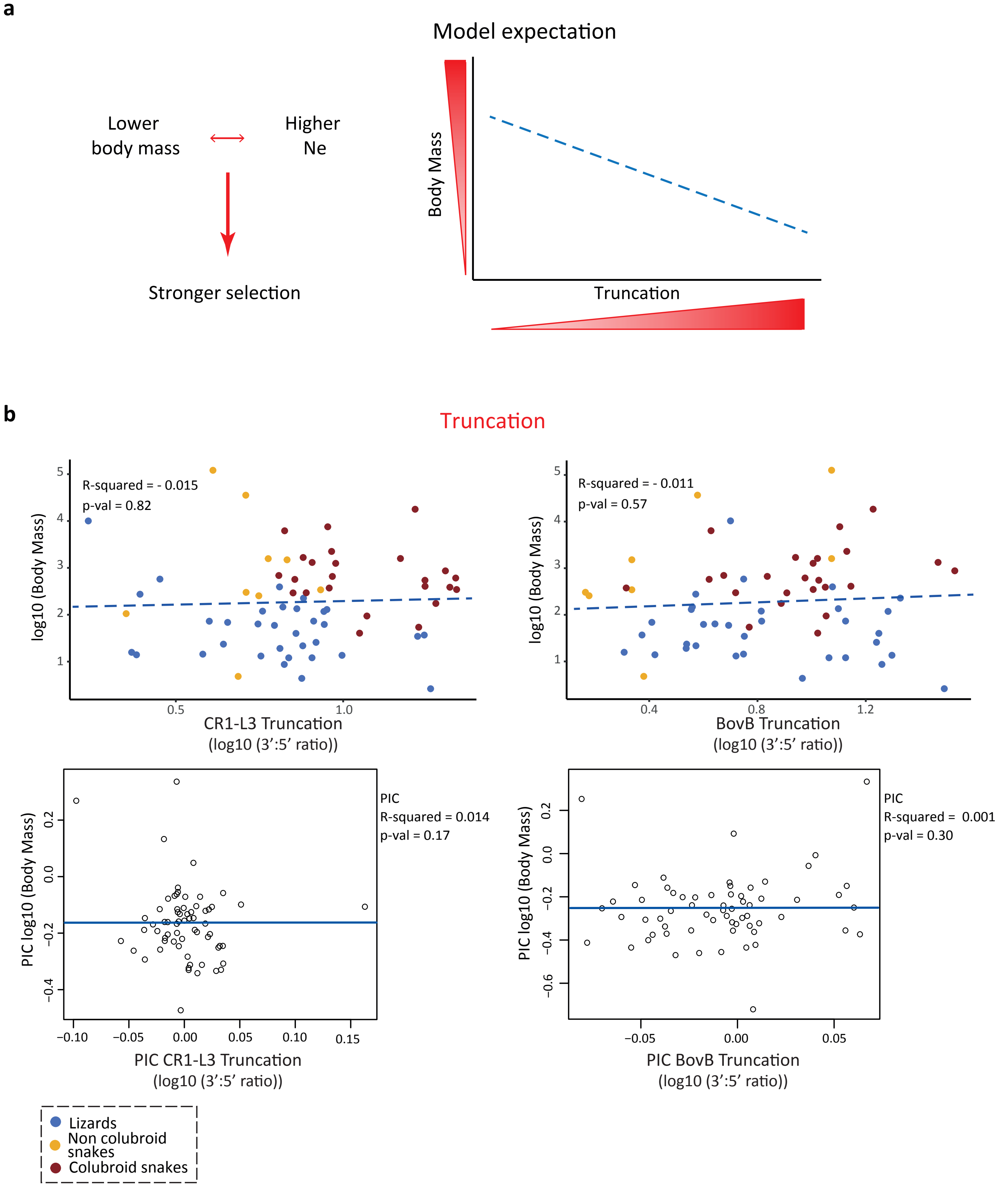

Supplementary Fig. 12 a and b. Relationship between adult body mass and truncation across 66 squamate species. a) Expected trends linking adult body mass (here used as proxy for effective population size) under a demographic explanation of the relationship between repeat element genomic abundance and TE copy length (truncation). b) Scatter plots and associated phylogenetically independent contrasts (PICs) show the lack of significant relationships between adult body mass and CR1-L3 (left) and BovB (right) truncation, which contrast with what would be expected under the demographic model. Truncation values were log-transformed prior to statistical tests to meet the assumption of normality.

c

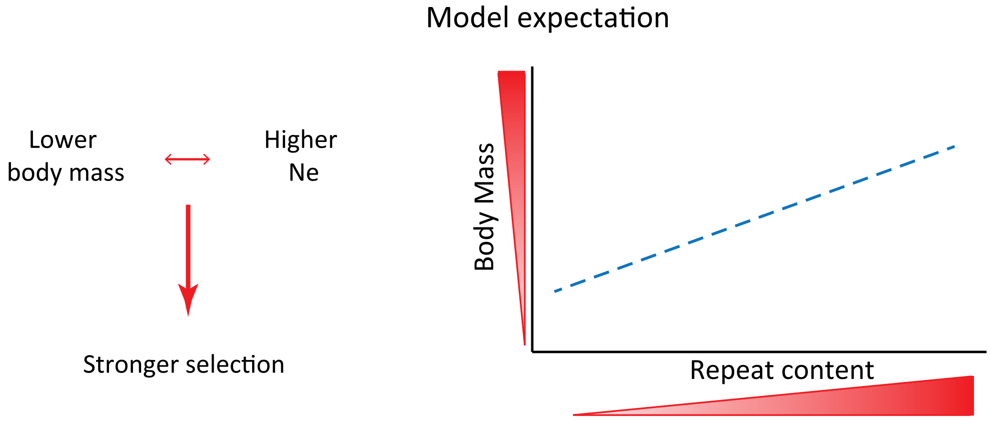

d

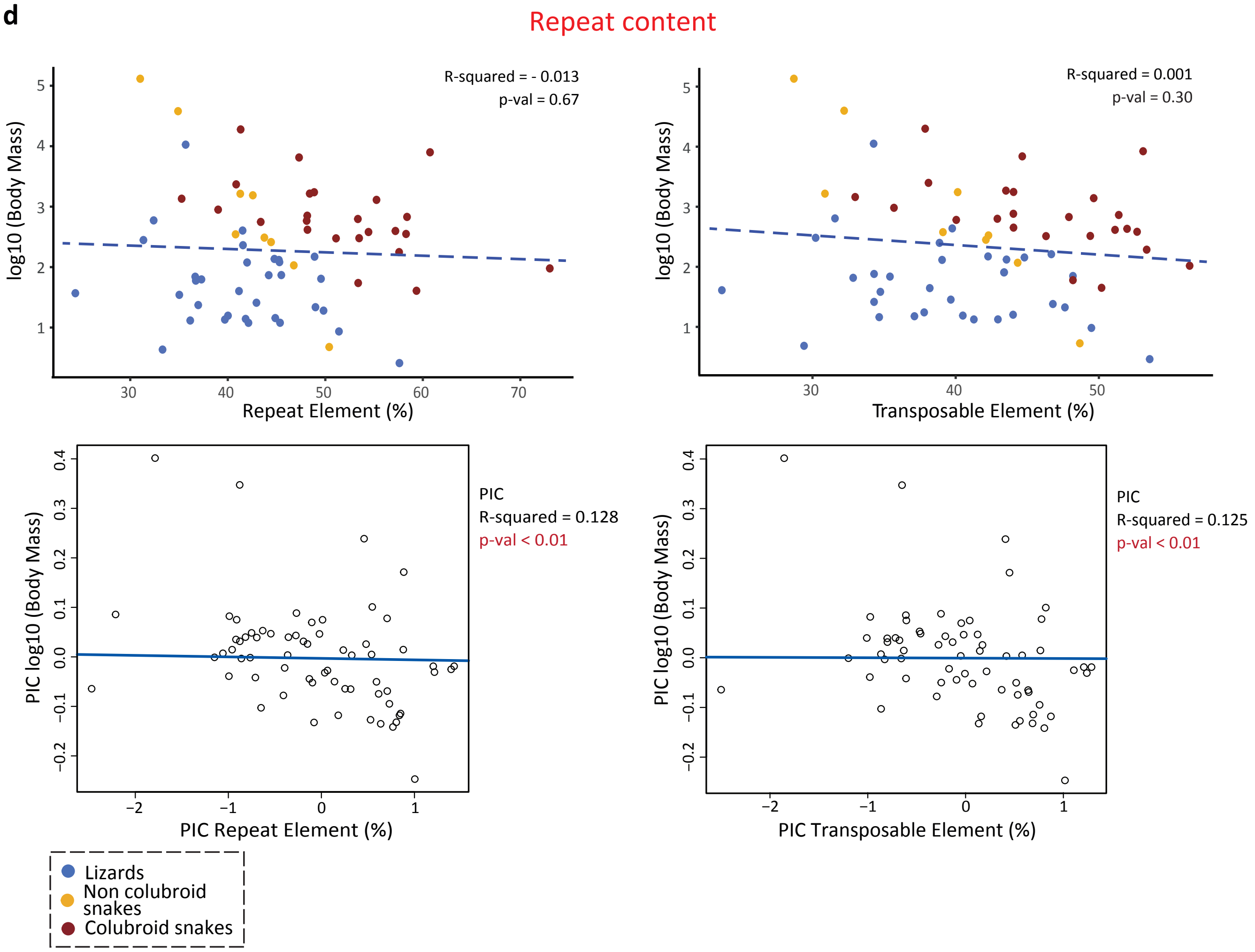

Supplementary Fig. 12 c and d. Relationship between adult body mass, repeat and TE content across 66 squamate species. c) Expected trends linking adult body mass (here used as proxy for effective population size) according to a demographic explanation of repeat element genomic abundance. d) Scatter plots and associated phylogenetically independent contrasts (PICs) show a significant relationship between adult body mass, total genomic repeat element content (left) and TE content (right), a trend opposite to what would be expected according to the model.

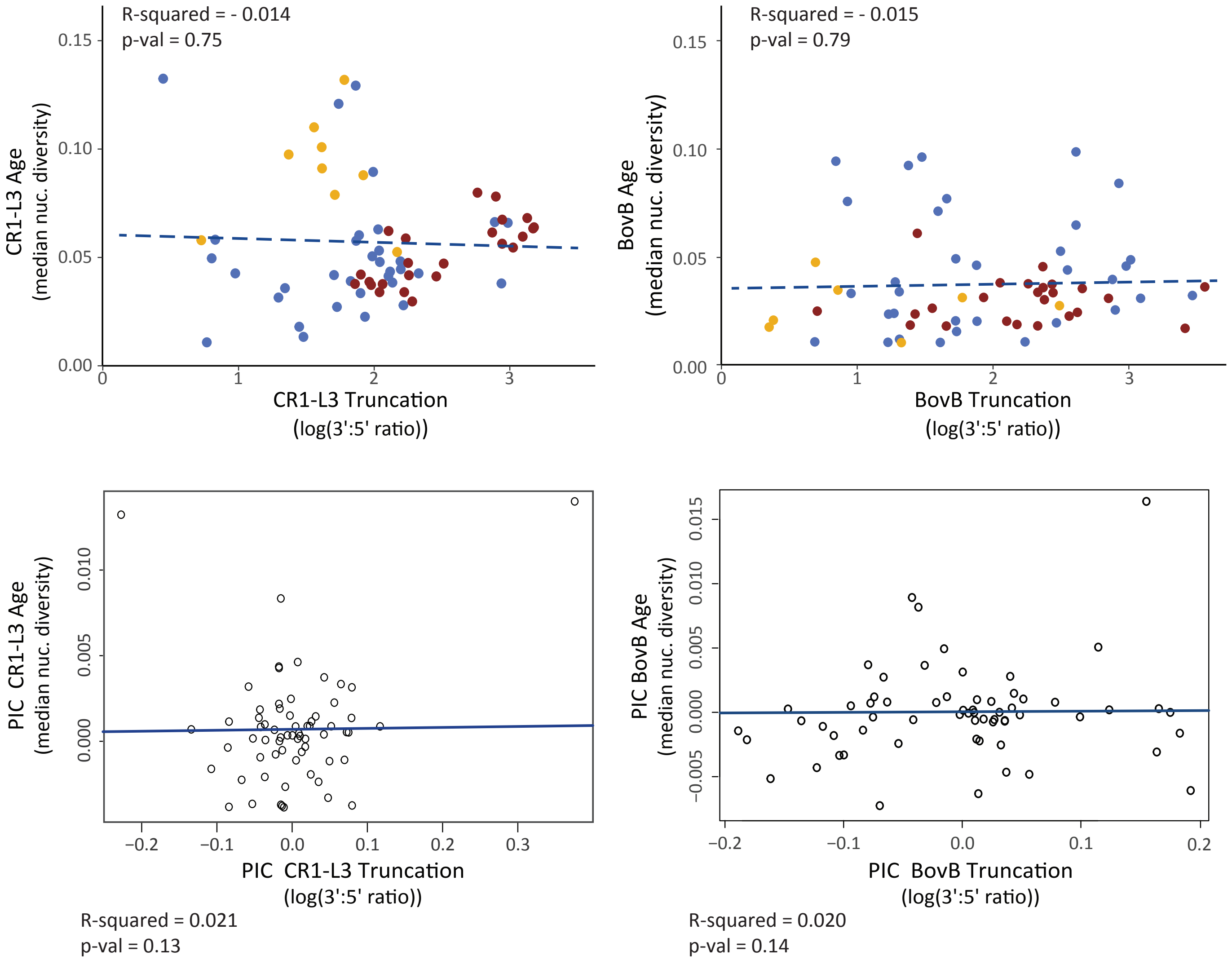

Supplementary Fig. 13. Relationship between median sequence divergence of CR1-L3 and BovB LINEs and their genomic abundance. Species-specific consensus sequences were used as reference to calculate estimates of nucleotide divergence (pairwise π) for all alignable CR1-L3 (left) and BovB (right) sequences. We excluded sites that appeared to define subfamilies prior to pairwise π estimation, and calculated relative frequency including only sequences with $\pi < 0.2$. Scatter plots (top) and associated phylogenetically independent contrasts (PICs, bottom) show no correlation between the median pairwise nucleotide diversity (used as a proxy for element age) and genomic abundance of CR1-L3 and BovB LINEs. Truncation values were log-transformed prior to performing statistical analyses to meet the assumption of normality. *Typhlops reticulatus* and *Anilius scytale* were not included in analyses of BovB LINEs (age data not retrievable).

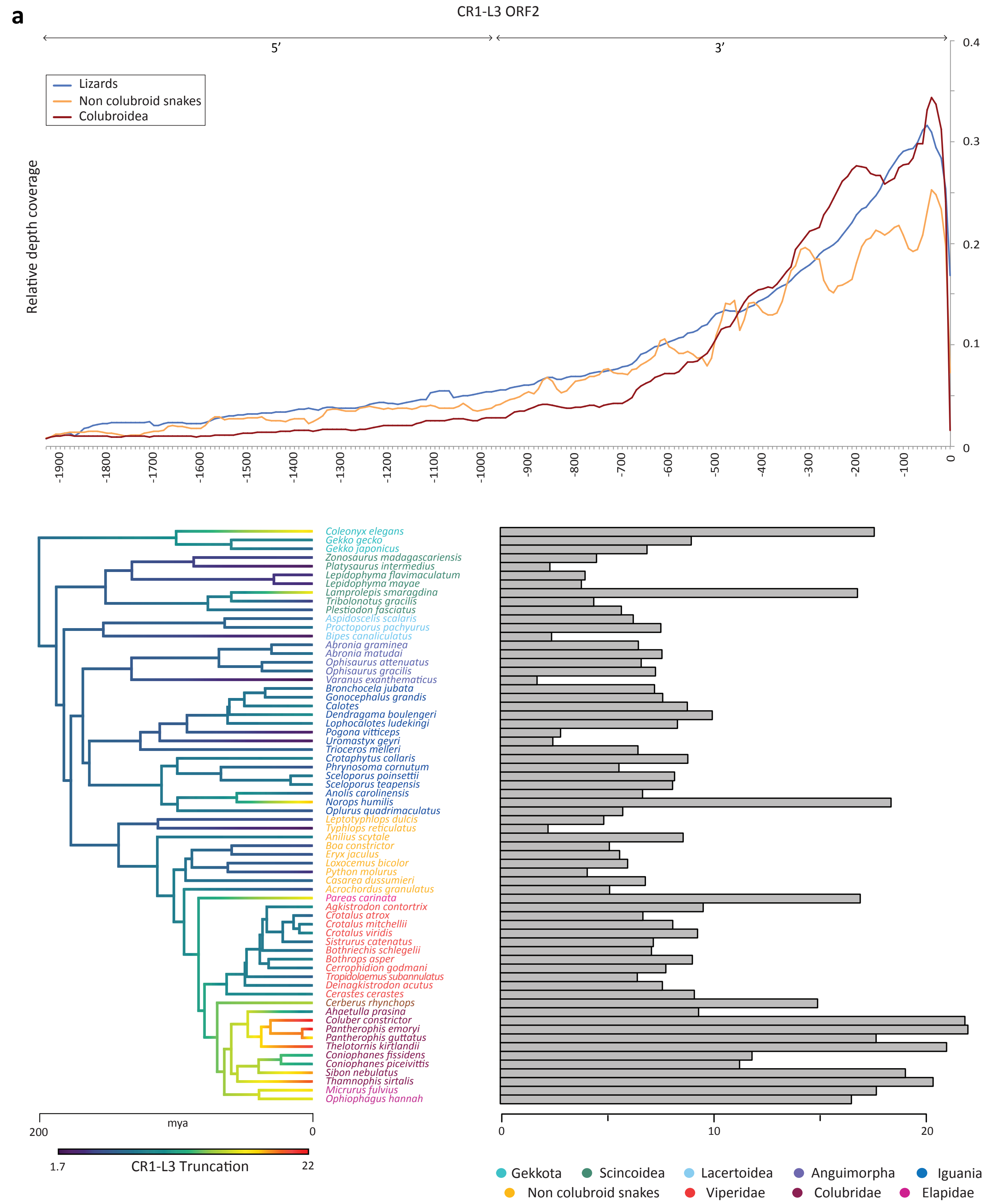

Supplementary Fig. 14a. Observed truncation patterns of CR1-L3 LINEs across 66 squamate species. Top: line graph shows the average relative coverage of mapped reads along the ORF2 of CR1-L3 LINE consensus sequences for each major squamate group. Position is reported relative to the 3'end of the reference. Bottom: horizontal bar plot reports the extent of truncation for each squamate species sampled, calculated as the ratio of read depth coverage of reads mapping to the second half (3') and to the first half (5') of the consensus. Branches on the time-calibrated phylogeny are colored accordingly the degree of truncation.

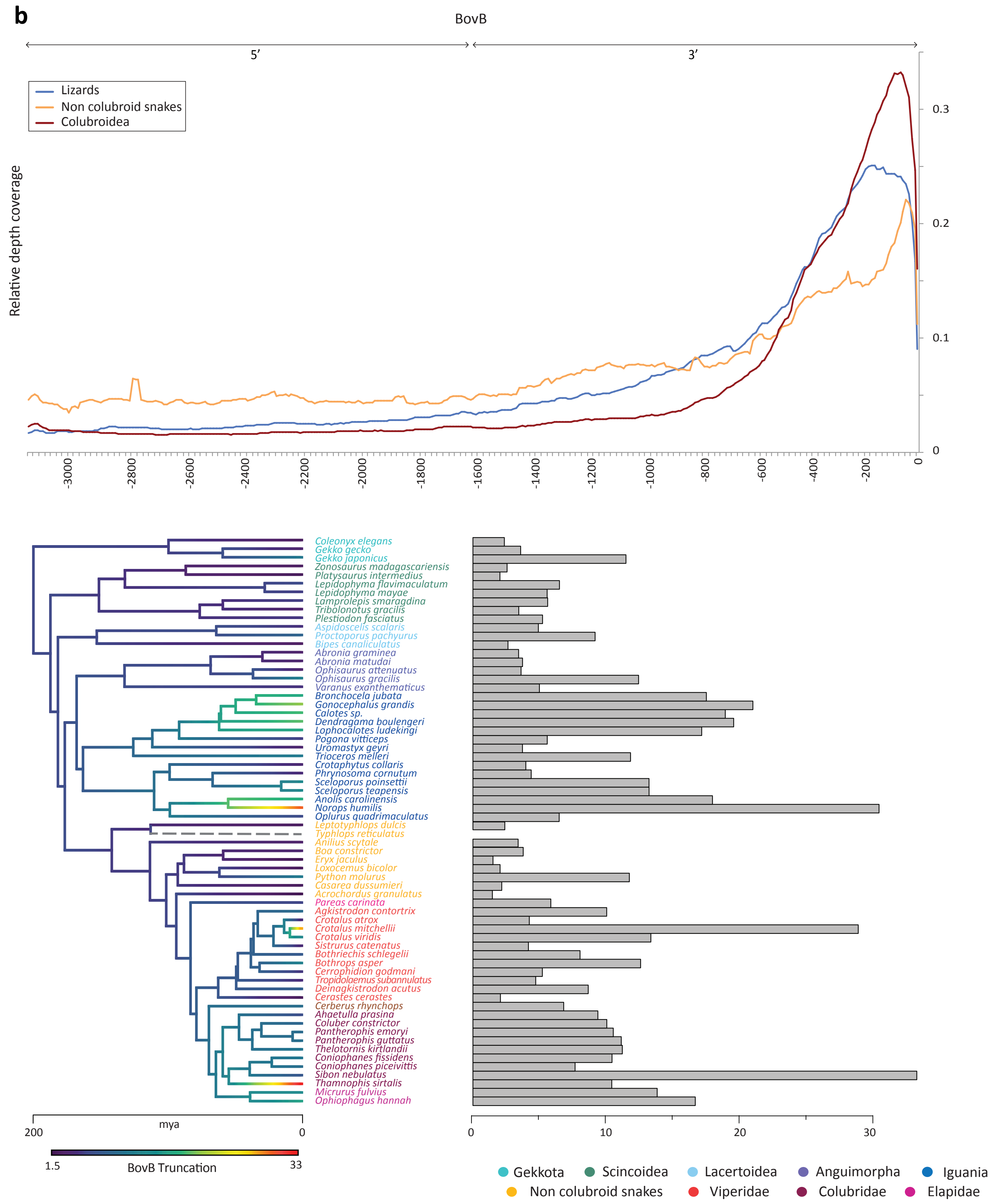

Supplementary Fig. 14b. Observed truncation patterns of BovB LINEs and lineage-specific evolutionary rates across 66 squamate species. Top: line graph shows the average relative coverage of mapped reads along BovB consensus sequences for each major squamate group. Position is reported relative to the 3'end of the reference. Bottom: horizontal bar plot reports the extent of truncation for each squamate species sampled, calculated as the ratio of read depth coverage of reads mapping to the second half (3') and to the first half (5') of the consensus. Branches on the time-calibrated phylogeny are colored accordingly the degree of truncation.

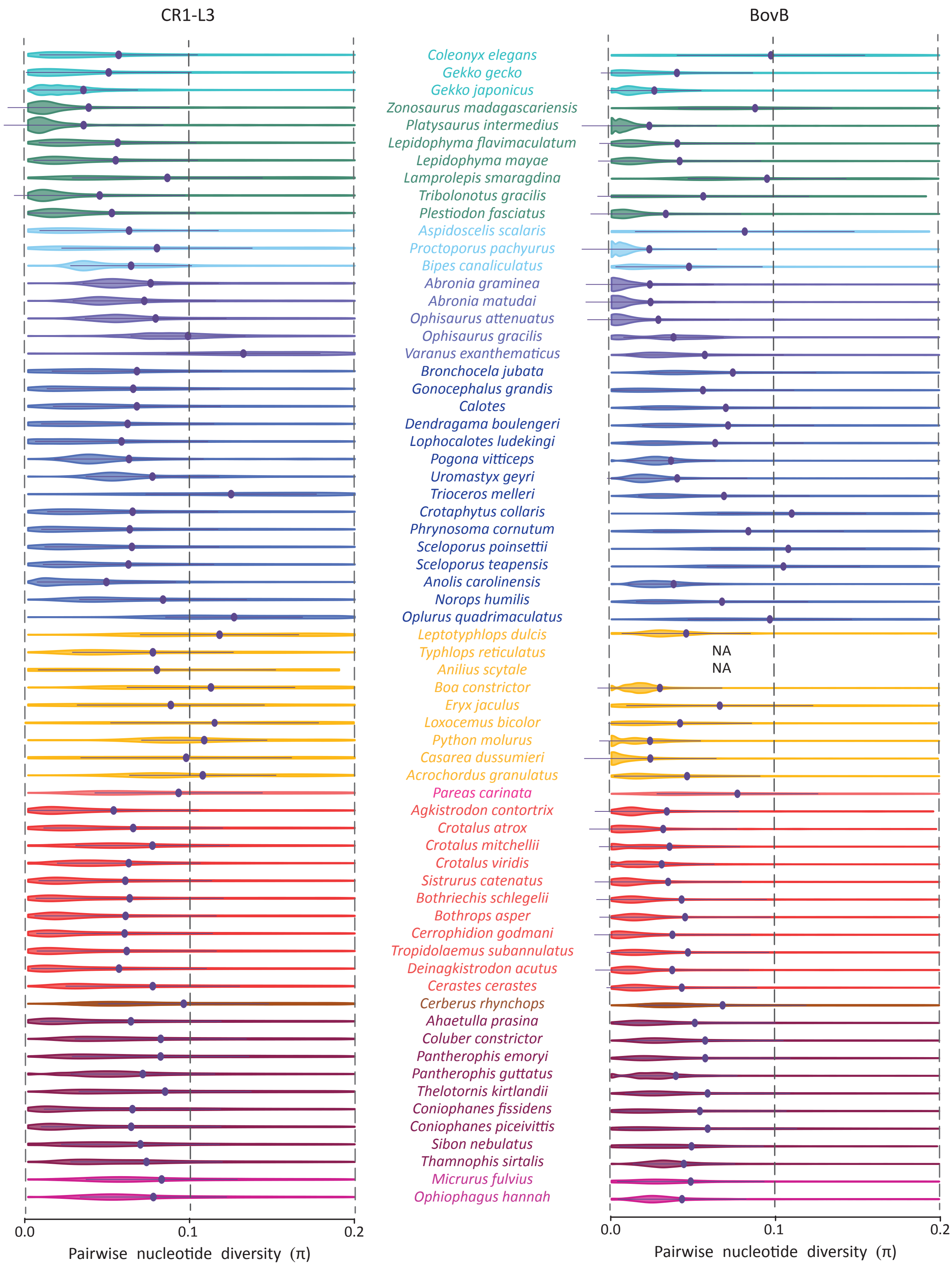

Supplementary Fig. 15. Estimated sequence divergence of CR1-L3 and BovB LINEs. Species-specific consensus sequences were used as reference to calculate estimates of divergence levels (pairwise π) for all alignable CR1-L3 (left) and BovB (right) sequences. We excluded sites that appeared to define subfamilies prior to pairwise π estimation, and calculated relative frequency including only sequences with π < 0.2. Violin plots show the empirical distribution of sequence divergence from the consensus for each species according to a kernel density estimation (KDE). Species with higher frequency of copies on the leftmost side of the violin plot are characterized by more recent amplification of the TE subfamily. Data that satisfies the filtering parameters are missing for *Typhlops reticulatus* and *Anilius scytale* BovBs.

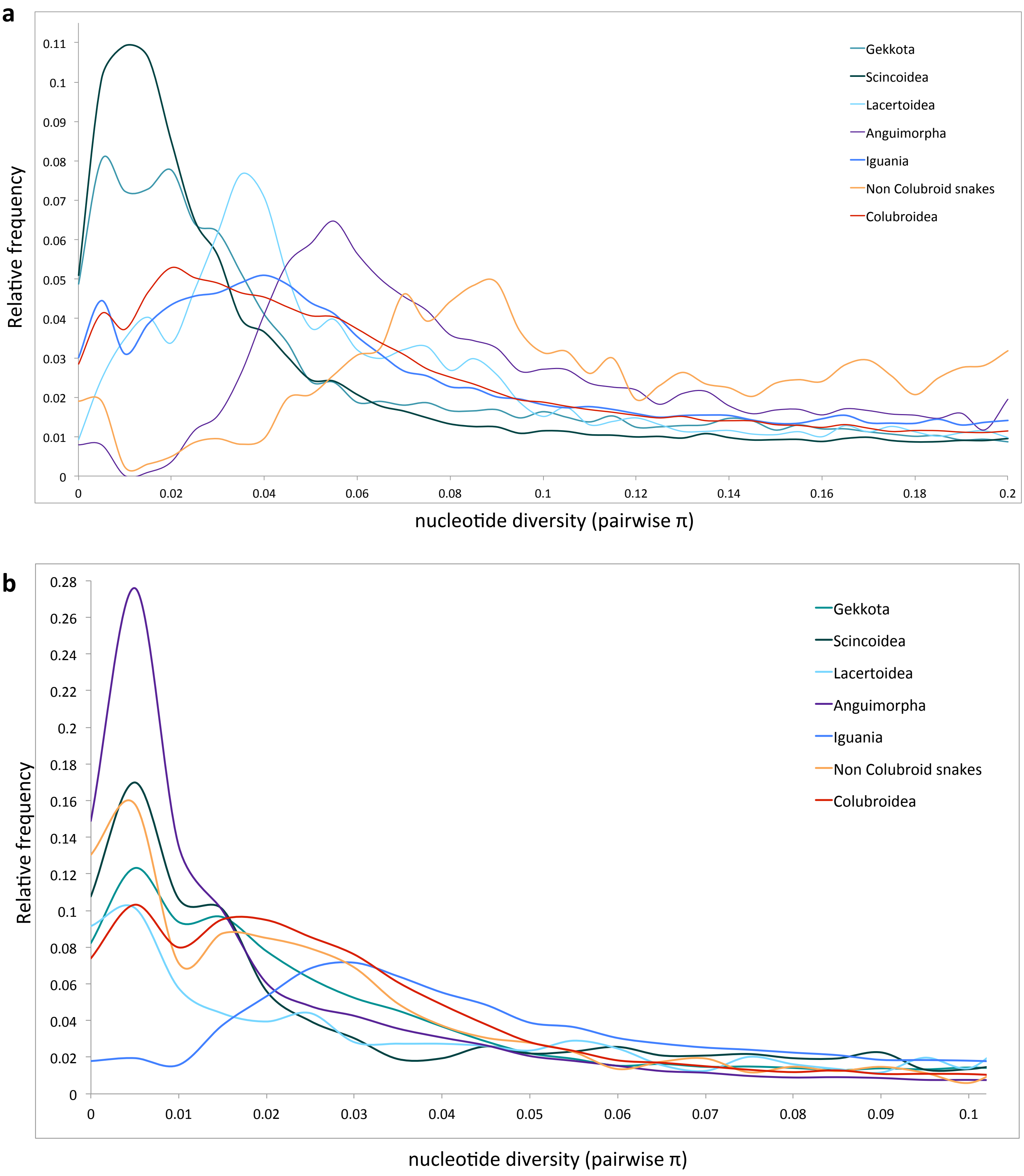

Supplementary Fig. 16. Estimated sequence divergence of CR1-L3 and BovB LINEs. Species-specific consensus sequences were used as reference to calculate estimates of divergence levels (pairwise π) for all alignable CR1 (a) and BovB (b) sequences. We excluded sites that appeared to define subfamilies prior to pairwise π estimation, and calculated relative frequency including only sequences with $\pi < 0.2$. Line graphs show the average sequence frequency distribution for each major squamate clade with detailed taxonomic organization of lizard species. Clades with higher relative frequency of copies on the leftmost side of the plot are characterized by a more recent amplification of the TE subfamily (e.g., Gekkota and Scincoidea for CR1-L3, and Anguimorpha for BovB).

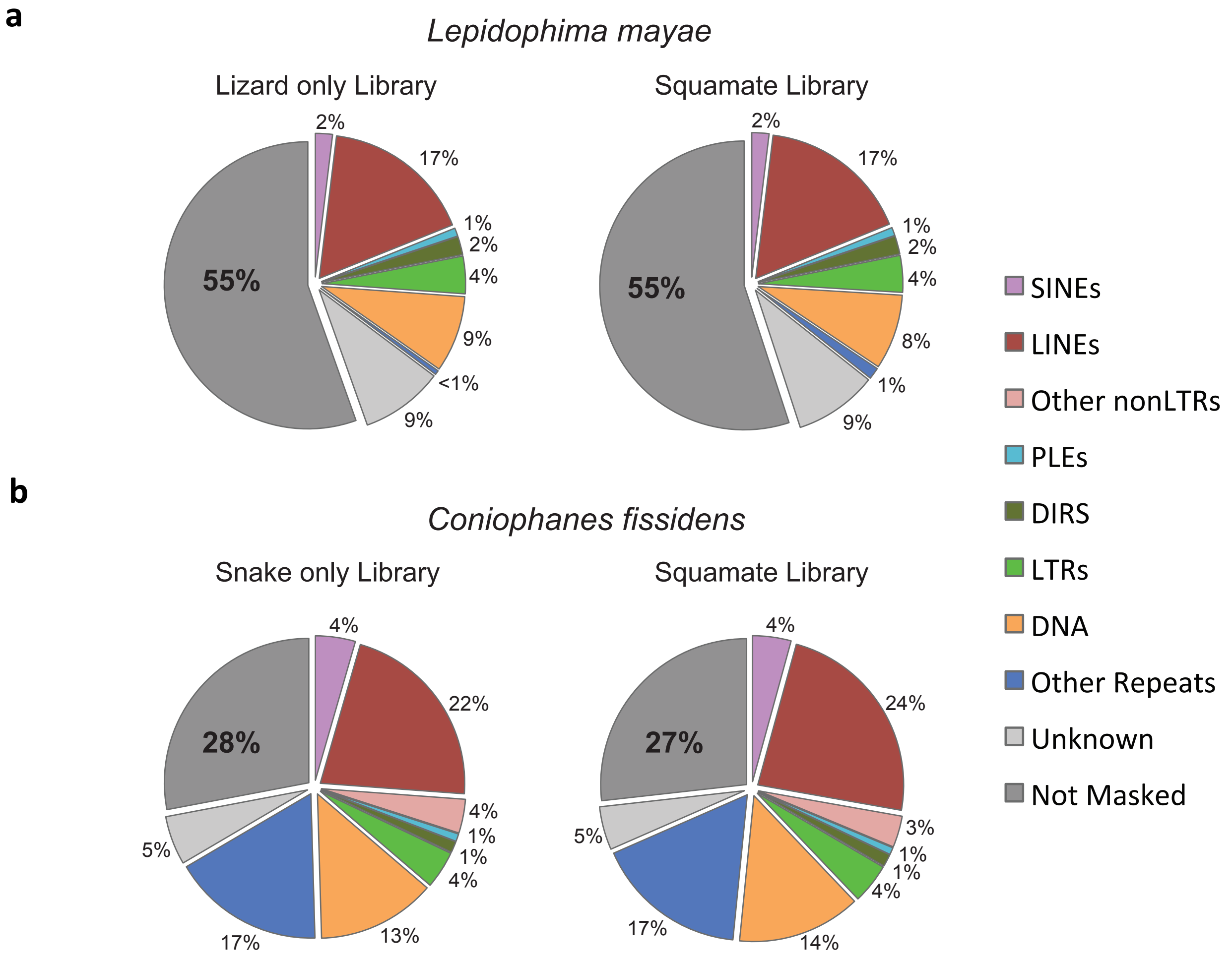

Supplementary Fig. 17. Masking strategy comparison for two squamate species. Pie charts report the repeat element genomic composition for (a) one lizard -*Lepidophyma mayae*- and (b) one snake -*Coniophanes fissidens*- species (randomly chosen for the comparative analysis). Charts on the left report masking results performed in RepeatMasker using a lineage specific repeat element reference library, whereas charts on the right reflect data generated using a single squamate repeat element reference library. Given that the two strategies brought to no difference in the masking quality, we consistently used the first approach (lineage-specific library) for within lineage consistency and optimization of RepeatMasker running time.

Supplementary Table 1. BEAST 2 priors used to estimate divergence times.
Clade ages for the Amniotes, Reptiles, Lepidosaurs, and Archosaurs were obtained from Benton and Donoghue 2007 [4], except for Alethinophidian snakes, Iguania, and Gekkota (Pyron et al. 2015 [5]).

Clade	Distribution	Mean	Sigma	Offset
**Reptile-Mammal Split**	Normal	321.3	4.64	0
**Archosaur-Lepidosaur Split**	Normal	277.8	11.2	0
**Squamate-Rhynchocephalia**	Log Normal	1	1	222.8
**Crocodile-Avian Split**	Normal	244.7	2.9	0
**Gekkota**	Normal	86.5	10	0
**Iguania**	Normal	146.4	5	0
**Alethinophidia**	Normal	102.75	4.625	0

Supplementary Table 2. Generation time assessments used for PSMC.

Species	Generation time	Source
Ophisaurus gracilis	2 years	Lindemann; Animal diversity web 2009 [6]
Pogona vitticeps	1-2 years	Pest risk assessment: Central bearded dragon (*Pogona vitticeps*) [7]
Boa constrictor	3 years	Lindemann; Animal diversity web 2009 [6]
Python molurus	2-3 years	Lindemann; Animal diversity web 2009 [6]
Crotalus mitchellii	3 years	Klauber; Rattlesnakes: Their Habits, Life Histories, and Influence on Mankind.[8]
Crotalus viridis	2-3 years	Lindemann; Animal diversity web 2009 [6]
Deinagkistrodon acutus	3-4 years	Lindemann; Animal diversity web 2009 [6]
Thamnophis sirtalis	1-2 years	Lindemann; Animal diversity web 2009 [6]

Supplementary References

1. Gregory TR. Animal Genome Size Database. (last accessed August 2017).

2. Smit AFA, Hubley R, Green P. RepeatMasker Genomic Datasets. (last accessed August 2017).

3. Kapusta A, Suh A, Feschotte C. Dynamics of genome size evolution in birds and mammals. *Proc Natl Acad Sci USA* **114**, E1460-E1469 (2017).

4. Benton MJ, Donoghue PCJ. Paleontological evidence to date the tree of life. *Mol Biol Evol* **24**, 26-53 (2007).

5. Pyron RA, Burbrink FT, Wiens JJ. A phylogeny and revised classification of Squamata, including 4161 species of lizards and snakes. *BMC Evol Biol* **13**, 93 (2013).

6. Animal Diversity Web. https://animaldiversity.org/accounts/Animalia/ (2017).

7. DPIPWE. Pest Risk Assessment: Central bearded dragon (Pogona vitticeps). Department of Primary Industries, Parks, Water and Environment. Hobart, Tasmania. (2011).

8. Klauber L. M. Rattlesnakes: their habits, life histories, and influence on mankind. *Univ Cal Press Berkeley and Los Angeles* **2** (1972).
