# Supplementary material for: Squamate reptiles challenge paradigms of genomic repeat element evolution set by birds and mammals
Source: Nat Commun. 2018 Jul 17;9:2774. doi: 10.1038/s41467-018-05279-1 (PMC6050309; doi:10.1038/s41467-018-05279-1)
Supplement: Supplementary file 3 — Description of Additional Supplementary Files [file 41467_2018_5279_MOESM3_ESM.pdf]

Description of Additional Supplementary Files

File Name: Supplementary Data 1
Description: Genome sampling statistics for 66 squamate species

File Name: Supplementary Data 2
Description: Flow cytometry estimates of squamate, bird and mammal genome size (C-value)

File Name: Supplementary Data 3
Description: Genomic transposable element content in bird and mammal genomes

File Name: Supplementary Data 4
Description: Repeat element landscape composition for 66 sampled squamate genomes estimated using RepeatMasker

File Name: Supplementary Data 5
Description: Microsatellite density estimates across 66 squamate species

File Name: Supplementary Data 6
Description: Statistics of the microsatellite landscape across lineages of squamate reptiles

File Name: Supplementary Data 7
Description: Statistical analysis of AATAG microsatellite loci seeding by transposable elements for 8 squamate genomes

File Name: Supplementary Data 8
Description: Adult body mass measurements for 66 sampled squamate species

File Name: Supplementary Data 9
Description: Multiple species alignment and phylogeny of BovB LINEs for 87 metazoa species or groups

File Name: Supplementary Data 10
Description: Multiple species alignment and phylogeny of CR1 LINEs for 155 vertebrate species or groups
